# Identification of mouse colony-forming endothelial progenitor cells for postnatal neovascularization: a novel insight highlighted by new mouse colony-forming assay

**DOI:** 10.1186/scrt168

**Published:** 2013-02-28

**Authors:** Shizu Tsukada, Sang-Mo Kwon, Takenori Matsuda, Seok-Yun Jung, Jun-Hee Lee, Sang-Hun Lee, Haruchika Masuda, Takayuki Asahara

**Affiliations:** 1 Department Regenerative Medicine, Tokai University of Medicine, Kanagawa, 259-1193, Japan; 2 Department of Physiology, School of Medicine, Medical Research Institute, Pusan National University Beomeo-ri, Mulgeum-eup, Yangsan, Gyeongsangnam-do, 626-870, Republic of Korea; 3 Kobe Institute of Biomedical Research and Innovation, 2-2 Minatojima-minamimachi, Chuo-ku, Kobe, Hyogo, 650-0047, Japan

## Abstract

**Introduction:**

Endothelial progenitor cells (EPCs) play a critical role in restoration of ischemic diseases. However, the actual status of EPC development and the mechanisms of EPC dysfunctions in patients with various ischemic diseases remain unknown.

**Methods:**

To investigate the detailed function of EPCs in experimental murine models, we have established an EPC colony forming assay (EPC-CFA) in murine EPCs. The abilities of murine EPCs in differentiation, adhesive capacity, proliferative potency, and transplantation *in vitro *and *in vivo *were then examined.

**Results:**

Peripheral blood mononuclear cells (PB-MNCs), bone marrow mononuclear cells (BM-MNCs) or bone marrow c-Kit^+^/Sca-1^+ ^lineage negative (BM-KSL) cells differentiated into two types of EPC colony forming units (EPC-CFUs), large sized EPC (large-EPC)-CFUs and small sized EPC (small-EPC)-CFUs. Gene expression analysis demonstrated that both EPC-CFU-derived cells expressed eNOS, Flk-1 and VE-cadherin, markers of endothelial cells (ECs), although the small-EPCs derived from small-EPC-CFU were higher in number and showed more immature features (higher population of KSL cells). Functionally, the large-EPCs derived from large-EPC-CFU had higher adhesive capacity but lower proliferative potency than small-EPCs, showing improved tubular forming capacity and incorporation potency into primary EC-derived tube formation. Importantly, hindlimb ischemia increased the frequencies of large-EPC-CFUs differentiated from PB-MNCs and bone marrow. Actually, transplantation of large-EPCs into ischemic hindlimb enhanced neovascularization in hindlimb ischemia model, although small-EPCs or murine ECs did not, suggesting that large-EPC-CFUs might play an important role in restoration of ischemic diseases.

**Conclusions:**

We demonstrated, using a murine ischemia model, that the EPC-CFA could be a useful way to investigate the differentiation levels of murine EPCs, further providing a crucial clue that large-EPC-CFU status may be more functional or effective EPCs to promote neovascularization.

## Introduction

Endothelial progenitor cells (EPCs) [1-3] play an important role in the restoration of ischemic vascular diseases [2-5]. Recently, several independent groups have shown that transplantation of EPCs into ischemic hindlimb or myocardial tissue improves organ function following the growth of new vessels [6-11]. In clinical aspects, the frequency of circulating EPCs may also serve as a biomarker for vascular function, and the number of circulating EPCs has been reported to be reduced in patients with diabetes mellitus or risk factors for coronary artery disease and to negatively correlate with the Framingham cardiovascular risk score [12-15]. However, the actual mechanical status of EPC development and the 'evaluation system' for EPC dysfunctions in patients with various ischemic diseases remain to be disclosed.

Because EPCs accumulate in ischemic injured tissues and repair injured tissue following cluster formation [1,2,9], not only the number of EPCs identified by uptake of acetylated-low density lipoprotein (acLDL) and lectin reactivity but also the colony-forming potential of EPCs is important for angiogenic therapy. Thus, the assay system in which colony-forming potential of EPCs can be assessed is important. EPCs should encompass a group of cells existing in a variety of stages, ranging from hemangioblastic hematopoietic stem cells to fully differentiated endothelial cells (ECs), and EPCs can be classified into stages according to differentiation levels in each circulating EPC and tissue EPC [16]. Recently, the methods to culture colony-forming unit-endothelial cells (CFU-ECs) [14] or to culture endothelial colony-forming cells (ECFCs) were established on mononuclear cells from peripheral blood or cord blood [17-20]. However, it was reported that CFU-ECs were not EPCs but were myeloid cells that differentiate into phagocytic macrophages and that T cells could mimic the morphology of CFU-ECs [19,21]. Besides, the culture of ECFCs enables us to evaluate the EPC colony-forming potential change as EPCs differentiated during culture *in vitro*. In these assay systems, each EPC at different differentiation levels could not be discriminated at the same time, and the differentiation capacities of immature stem cells could not be tested. In our laboratory, EPC-CFA, a novel method to assess the colony-forming potential of EPCs at different differentiation levels, was recently established and enables us to investigate the commitment of each cell [22-24].

In the present study, we aimed to methodologically establish the murine EPC-CFA on PB-MNCs, BM-MNCs, or BM-KSL cells by analyzing the functions of each EPC-CFU at different differentiation levels and to clarify the roles of each EPC-CFU at different differentiation levels *in vivo *by using hindlimb ischemic mice. By EPC-CFA, we investigated the status of EPC differentiation in response to ischemic signals and the effects of two types of EPC-CFUs - small-EPC-CFUs or large-EPC-CFUs - in a hindlimb ischemia model on *in vivo *neovascularization.

## Materials and methods

### Animals

Experiments were performed on male 8- to 10-week-old C57BL/6J mice and BALB/CA-nu/nu mice (Japan Clea, Tokyo, Japan) maintained under a 12-hour light/dark cycle and in accordance with the regulations of Tokai University. Standard laboratory chow and water were available *ad libitum*. The protocols were approved by guidelines of the Institutional Animal Care and Use Committee of the Isehara Campus, Tokai University School of Medicine, based on the Guide for the Care and Use of Laboratory Animals (National Research Council) (Institutional Review Board ID number 083005).

### Preparation

Peripheral blood was obtained from the heart immediately before sacrifice and was separated by Histopaque-1083 (Sigma-Aldrich, St. Louis, MO, USA) density gradient centrifugation, as previously described [25]. Briefly, low-density mononuclear cells were harvested and washed twice with Dulbecco's phosphate-buffered saline (PBS) supplemented with 2 mmol/L ethylenediaminetetraacetic acid (EDTA). Contaminated red blood cells were hemolyzed by using ammonium chloride solution. BM-MNCs were obtained by flushing the femurs and tibias and reacted with a mixture of biotinylated monoclonal antibodies against B220 (RA3-6B2), CD3 (145-2C11), CD11b (M1/70), TER-119 (Ly-76), and Gr-1 (RB6-8C5) (all from BD Pharmingen, San Diego, CA, USA) as lineage markers to deplete lineage-positive cells from BM-MNCs by using AutoMACS (Becton Dickinson, Franklin Lakes, NJ, USA). Lineage-negative bone marrow cells (BM-LNneg) were incubated with saturating concentrations of directly labeled anti-c-Kit (at 1:25 dilution) (BD Biosciences, Franklin Lakes, NJ, USA) and anti-Sca-1 antibodies (at 1:25 dilution) (BD Biosciences) for 30 minutes on ice, and then the c-Kit^+^/Sca-1^+ ^lineage-negative cells (BM-KSL) were isolated with live sterile cell sorting (FACSVantage SE; Becton Dickinson).

### Endothelial progenitor cell colony-forming assay

Various cells were cultured in methylcellulose-containing medium M3236 (StemCell Technologies, Vancouver, BC, Canada) with 20 ng/mL stem cell-derived factor (Kirin, Tokyo, Japan), 50 ng/mL vascular endothelial (VE) growth factor (R&D Systems, Minneapolis, MN, USA), 20 ng/mL interleukin-3 (Kirin), 50 ng/mL basic fibroblast growth factor (Wako, Osaka, Japan), 50 ng/mL epidermal growth factor receptor (Wako), 50 ng/mL insulin-like growth factor-1 (Wako), 2 U/mL heparin (Ajinomoto, Tokyo, Japan), and 10% fetal bovine serum (FBS) on a 35-mm dish for 8 days. Cell densities for each sample were as follows: PB-MNCs 7 × 10^5 ^cells per dish, BM-MNCs 1 × 10^4 ^cells per dish, BM-LNneg 2.5 × 10^3 ^cells per dish, and BM-KSL 500 cells per dish. The EPC-CFUs were identified as large-EPC-CFUs or small-EPC-CFUs by visual inspection with an inverted microscope under 40× magnification. Large-EPC-CFUs were composed of spindle-shaped cells, and small-EPC-CFUs were composed of round adhesive cells.

### Endothelial progenitor cell-colony-forming unit staining

After 8 days in culture, the EPC-CFU cultures were treated with 0.4 μg/mL 1,1'-dioctadecyl-3,3,3',3-tetramethyl-indocarbocyanine perchlorate-labeled acLDL (acLDL-DiI; Biomedical Technologies Inc., Stoughton, MA, USA) for 1 hour and fixed by application of 1 mL of 2% paraformaldehyde (PFA) for 1 hour at room temperature. After a wash of the methylcellulose-containing medium with PBS, the cultures were reacted with fluorescein isothiocyanate (FITC)-conjugated BS-1 lectin (Sigma-Aldrich) for 1 hour at room temperature. After a wash with PBS, the cultures were observed under a fluorescence microscope (IX70; Olympus, Tokyo, Japan).

### Large-endothelial progenitor cell or small-endothelial progenitor cell isolation

Cells composed of small-EPC-CFUs were collected with a pipette under a microscope as small-EPCs. Then the cultures were washed with PBS, and large-EPCs were harvested after treatment with 2 mmol/L EDTA/PBS. For the purpose of cell transplantation into a hindlimb ischemia model, non-attached cells were isolated as small-EPCs by washing with PBS, whereas attached cells were harvested as large-EPCs by treatment with EDTA/PBS (5 mmol/L) for 5 minutes at 37°C.

### Adhesive assay

Culture plates (24-well) were coated with human fibronectin (100 μg/mL; Gibco, now part of Invitrogen Corporation, Carlsbad, CA, USA). Large-EPCs or small-EPCs (2 × 10^4 ^cells per well) were allowed to attach in EGM-2 (Cambrex Bio Science Walkersville, Walkersville, MD, USA) for 20 minutes at 37°C, and the non-adherent cells were aspirated. The adherent population was fixed with 1% PFA for 20 minutes and stored in PBS. The numbers of adherent cells were quantified from counts in six random microscopic fields per well.

### Proliferation assay

At day 7, EPC-CFU cultures were treated with 10 μmol/L bromodeoxyuridine (BrdU) (Sigma-Aldrich) and incubated for 24 hours. BrdU positivities of large-EPCs or small-EPCs were analyzed by using BrdU flow kits (BD Pharmingen) and a fluorescence-activated cell sorter, as previously described [26].

### Tubular formation assay

Two-week derived CD133^- ^mononuclear cells of human cord blood were used as ECs. These cells were confirmed to be ECs by tubular formation and immunocytochemistry of endothelial nitric oxide synthase (eNOS), kinase insert domain receptor (KDR), and VE-cadherin (data not shown) [5]. Each small-EPC or large EPC was labeled with acLDL-DiI for 1 hour. After washing of the labeled small-EPCs or large-EPCs with PBS, the 1 × 10^3 ^cells were mixed together with 1.2 × 10^4 ^ECs in 50 μL of 2% FBS/EBM-2. Cell suspension (50 μL) was applied onto 50 μL of Matrigel (BD, Franklin Lakes, NJ, USA) per well of a 96-well plate (BD Falcon, Franklin Lakes, NJ, USA) and then incubated for 8 hours. After incubation, the numbers of tubular formation were counted on a display of Photoshop software (Adobe, San Jose, CA, USA) after a picture per well was taken at 40× magnification under a light microscope (Eclipse TE300; Nikon, Tokyo, Japan). The numbers of incorporated labeled cells into tubes were also counted on a display of Photoshop software after a picture per well was taken at 100× magnification under a fluorescence microscope.

### Secondary culture

Isolated small-EPCs (5 × 10^4^) were suspended in 50 μL of Iscove's modified Dulbecco's medium (IMDM) (Gibco) and applied onto 100 μL of methylcellulose-containing medium per well of a 96-well plate (BD Falcon). After 2 days of incubation, methylcellulose-containing medium was changed to IMDM containing acLDL-DiI and BS-1 lectin-conjugated FITC and then incubated for 1 hour. After a wash with PBS, cultures were observed under a fluorescence microscope.

### Reverse transcription-polymerase chain reaction

Total RNA of small-EPCs or large-EPCs was prepared with an RNeasy Micro/Mini kit (Qiagen, Valencia, CA, USA). Reverse transcription-polymerase chain reaction (RT-PCR) was performed by using Superscript III Reverse Transcriptase (Invitrogen Corporation) with 1 μg of total RNA. PCR amplification was then performed with synthetic gene-specific primers for eNOS (forward primer, 5'-GGATTGTGTCACTTCGTTCGGT-3'; reverse primer, 5'-CAGCAGGATGCCCTAACTACCA-3'; product length, 183 base pairs (bp)), Flk-1 (forward primer, 5'-AAAGAGAGGAACGTCGGCAGA-3'; reverse primer, 5'-AAGCACACAGGCAGAAACCAGT-3'; product length, 376 bp), VE-cadherin (forward primer, 5'-AGATTCACGAGCAGTTGGTCA-3'; reverse primer, 5'-GATGTCAGAGTCGGAGGAATT-3'; product length, 355 bp), and β-actin (forward primer, 5'-AACACCCCAGCCATGTACGTA-3'; reverse primer, 5'-AAGGAAGGCTGGAAAAGAGCC-3'; product length, 416 bp) by using exTaq polymerase (Takara, Kyoto, Japan). To quantify transcripts, semi-quantitative RT-PCRs were performed and normalized to Actb, which encodes β-actin. PCRs were performed at 94°C for 45 seconds, 64°C for 1 minute, and 72°C for 1 minute for 35 or 33 or 22 cycles and analyzed on 2% agarose gels.

### Flow cytometry

For flow cytometry analysis, we used monoclonal antibodies specific to Sca-1 and c-Kit. BM-LNneg- or EPC-CFU-derived cells were incubated with directly labeled anti-Sca-1 (at 1:100 dilution) and anti-c-Kit (at 1:100 dilution) antibodies for 30 minutes on ice. The cells were analyzed by two-color flow cytometry by using a FACS caliber (Becton Dickinson).

### Animal model of ischemic hindlimb

Unilateral hindlimb ischemia was created in C57BL/6J mice or BALB/CA-nu/nu as previously described [27]. Briefly, the animals were anesthetized with Nembutal (60 mg/kg intraperitoneally; Dainippon Sumitomo Pharma Co., Osaka, Japan) and then an incision in the skin overlying the middle portion of the left hindlimb was performed. After ligation of the proximal end of the femoral artery, the distal portion of the saphenous artery was ligated and the artery, as well as all side branches, was dissected free and excised. The skin was closed by using a surgical stapler.

### Monitoring of hindlimb blood flow

After anesthesia, hindlimb perfusion was measured by using a laser Doppler perfusion imaging system (LDPI; Moor Instruments, Wilmington, DE, USA). The stored perfusion values behind the color-coded pixels representing the microvascular blood flow distribution are available for analysis. Color photographs were recorded and analysis performed by calculating the average perfusion of the ischemic and non-ischemic foot. To account for variables such as ambient light and temperature, the results are expressed as the ratio of perfusion in the left (ischemic) versus right (normal) limb. In the EPC transplantation experiment, isolated small-EPCs, large-EPCs, or murine ECs (2.5 × 10^5^) derived from the aorta of C57BL/6J were transplanted into hindlimb induced nude mice by intramuscular injection, respectively (*n *= 8).

### Measuring of capillary density

Twenty-eight days after ischemia, capillary density was determined in tissue sections from the lower calf muscles of ischemic and healthy limbs by expressed as number of CD31^+ ^cells as ECs per myocyte. To stain the capillary, we performed a staining procedure with rat anti-mouse CD31 antibodies (BD Biosciences) or Alexa-fluor 594 (Molecular Probes, now part of Invitrogen Corporation) anti-iso-lectin B4 reagents (Sigma-Aldrich).

### Statistical analysis

All data were presented as mean ± standard deviation. *P *values were calculated by using the unpaired Student *t *test. For the analysis of *in vivo *ischemia experiments, the Scheffe's test was performed for the multiple comparisons after analysis of variance between each group. A *P *value of less than 0.05 was considered statistically significant.

## Results

### Development of murine endothelial progenitor cell colony-forming assay

**Figure 1:**
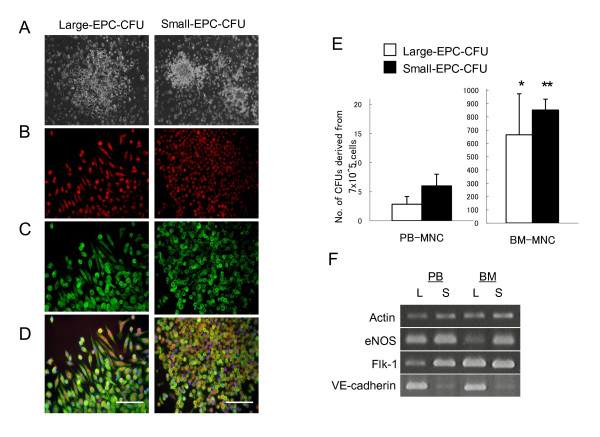
**Murine endothelial progenitor cell colony-forming units (EPC-CFUs)**. **(a) **Representative micrographs of large-EPC-CFUs or small-EPC-CFUs cultured from bone marrow mononuclear cells (BM-MNCs) for 8 days. Large-EPC-CFUs and small-EPC-CFUs were defined according to cell morphology as spindle-shaped cells or round cells, respectively. **(b-d) **EPC-CFUs were identified as double-positive cells due to 1,1'-dioctadecyl-3,3,3',3-tetramethyl-indocarbocyanine perchlorate-labeled acetylated-low density lipoprotein (acLDL-DiI) uptake (red) and BS-1 lectin reactivity (green). Scale bar represents 100 μm. **(e) **EPC colony-forming assay in murine peripheral blood mononuclear cells (PB-MNCs) or BM-MNCs. The frequencies of large-EPC-CFUs (white columns) or small-EPC-CFUs (black columns) from PB-MNCs or BM-MNCs (7 × 10^5 ^cells) were counted after 8 days of culture. *P < 0.05, **P < 0.01 versus PB-MNC-derived EPC-CFUs. **(f) **Expression patterns of endothelial nitric oxide synthase (eNOS), Flk-1, and vascular endothelial (VE)-cadherin genes in large-EPCs (L) or small-EPCs (S) from PB-MNCs or BM-MNCs. Both EPC-CFU-derived cells expressed markers of endothelial cells.

To address the detail functions and actual status of *in vivo *EPCs, we have first established a novel EPC-CFA in murine EPCs. After culture of PB-MNCs, BM-MNCs, or BM-KSL of C57BL/6J mice in growth factor-containing methylcellulose medium, these primitive cells differentiated into two types of EPC colony clusters: large-EPC-CFUs and small-EPC-CFUs (Figure 1a, data not shown). Morphologically, these cells are large-EPC-CFUs, which were composed mainly of spindle/round-shaped cells, whereas cells composed of small-EPC-CFUs were round. Both EPC-CFUs differentiated from primary PB-MNCs or primary BM-derived cells were identified as EPCs by acLDL uptake and BS-1 lectin reactivity, a typical feature of characterization of endothelial lineage cells (Figure 1b-d, data not shown). The frequencies of large-EPC-CFUs or small-EPC-CFUs differentiated from 7 × 10^5 ^PB-MNCs were 2.8 ± 1.3 and 6.0 ± 2.0 per dish, respectively. The normalized frequencies of large-EPC-CFUs or small-EPC-CFUs differentiated from 7 × 10^5 ^BM-MNCs were 665 ± 309 and 852 ± 82 per dish, respectively (Figure 1e). These results revealed that BM-MNCs had higher EPC colony-forming capacity than PB-MNCs. In this EPC-CFA, EPCs from primary murine cells could be classified into two types of EPC-CFUs and the colony-forming potential could be assessed by the frequency of EPC-CFUs. To check the commitment of each EPC-CFU-derived cell, eNOS, Flk-1, and VE-cadherin, markers of ECs, were examined. Gene expression profiles revealed that large-EPCs and small-EPCs expressed eNOS, Flk-1, and VE-cadherin gene in both PB-MNCs and BM-MNCs (Figure 1f), showing that large-EPCs strongly expressed VE-cadherin, a typical EC marker, although small-EPCs also expressed eNOS or Flk-1, each of which is a committed marker of endothelial lineage cells.

### Characterization of large endothelial progenitor cells or small endothelial progenitor cells

**Figure 2:**
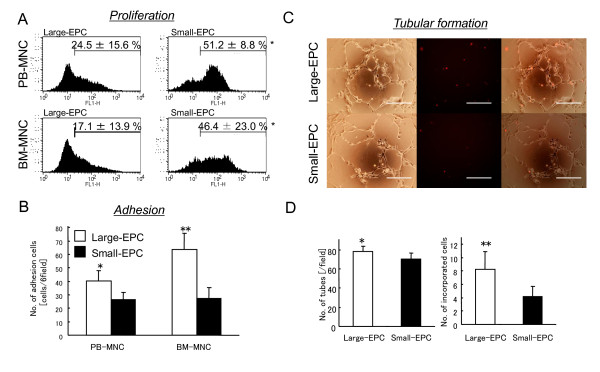
**Characterization of large-EPCs or small-EPCs**. **(a) **Proliferation assay of large-EPCs or small-EPCs from peripheral blood mononuclear cells (PB-MNCs) (upper) or bone marrow mononuclear cells (BM-MNCs) (bottom). After 7 days of culture, large-EPC-CFUs or small-EPC-CFUs were allowed to incorporate bromodeoxyuridine (BrdU) for 24 hours and analyzed by flow cytometry. Large-EPCs had significantly lower proliferative potency than small-EPCs in both PB-MNCs and BM-MNCs (*P < 0.05 versus large-EPCs). **(b) **Adhesion assay of large-EPCs or small-EPCs from PB-MNCs or BM-MNCs. Large-EPCs (white columns) or small-EPCs (black columns) were allowed to adhere to a fibronectin-coated plate for 20 minutes. More large-EPCs had adhesive capacity than small-EPCs. *P < 0.05, **P < 0.01 versus small-EPCs. **(c) **Tubular formation assay of large-EPCs or small-EPCs from BM-MNCs. Large-EPCs or small-EPCs labeled with 1,1'-dioctadecyl-3,3,3',3-tetramethyl-indocarbocyanine perchlorate-labeled acetylated-low density lipoprotein (acLDL-DiI) (red) were cocultured with endothelial cells (ECs) to form tubular structures within Matrigel. Representative light and fluorescent micrographs of ECs cocultured with large-EPCs (upper) and small-EPCs (bottom) are shown. Scale bar represents 500 μm. **(d) **Quantification of the number of tubes (left). Large-EPCs made a substantial contribution to tubular networks with ECs. *P < 0.05 versus small-EPCs. (d) Quantification of the number of cells incorporated into tubes (right). Small-EPCs showed minimal incorporation into the developing vascular network. **P < 0.01 versus small-EPCs. EPC, endothelial progenitor cell.

To characterize these two types of EPC-CFUs (large-EPC-CFUs or small-EPC-CFUs), we separately collected EPC-CFU-derived cells and investigated the functions of both EPC-CFUs. To determine the proliferation potency of each EPC-CFU-derived cell, we performed a proliferation assay. In PB-MNCs-derived EPC-CFUs, 24.5% ± 15.6% of large-EPCs and 51.2% ± 8.8% of small-EPCs incorporated BrdU. In BM-MNCs-derived EPC-CFUs, 17.1% ± 13.9% of large-EPCs and 46.4% ± 23.0% of small-EPCs incorporated BrdU (Figure 2a). More small-EPCs incorporated BrdU than large-EPCs, suggesting that large-EPCs have lower proliferation potency than small-EPCs. From observation of EPC-CFUs under a microscope, small-EPC-CFUs were constituted of more cells than large-EPC-CFUs and the areas of small-EPC-CFUs were significantly larger than those of large-EPC-CFUs (data not shown). We next defined an adhesive capacity of these two types of EPC-CFUs. The numbers of adherent large-EPCs or small-EPCs from PB-MNCs were 40.5 ± 7.6 and 26.3 ± 5.6 per field, respectively, and those from BM-MNCs were 63.7 ± 12.0 and 27.2 ± 8.0 per field, respectively (Figure 2b), proving that the large-EPCs have higher adhesive capacity than small-EPCs by 1.5-fold in PB-MNCs and 2.3-fold in BM-MNCs. To check tube-forming ability, large-EPCs or small-EPCs derived from BM were labeled with acLDL-DiI and cocultured with ECs, which were 2-week derived CD133^- ^mononuclear cells of human cord blood, on Matrigel. Fluorescent tagging of each EPC-CFU-derived cell with DiI enabled delineation from ECs (Figure 2c). The number of tubes in coculture with large-EPCs increased significantly compared with small-EPCs (large-EPCs; 78.3 ± 5.8, small-EPCs; 70.7 ± 8.4) (Figure 2d, left). Moreover, more large-EPCs were incorporated into tubes compared with small-EPCs (large-EPCs; 8.3 ± 2.7, small-EPCs; 4.2 ± 1.7) (Figure 2d, right), implying that large-EPCs made a substantial contribution to tubular networks with ECs, although small-EPCs showed minimal incorporation into the developing vascular network. Taken together, three independent results strongly indicated that large-EPCs and small-EPCs had different functions and that large-EPCs might be more mature EPCs with respect to adhesion ability and functional contribution of tubule networks of ECs.

### Importance of small-endothelial progenitor cells as large-endothelial progenitor cell-colony-forming unit sprouting cells

**Figure 3:**
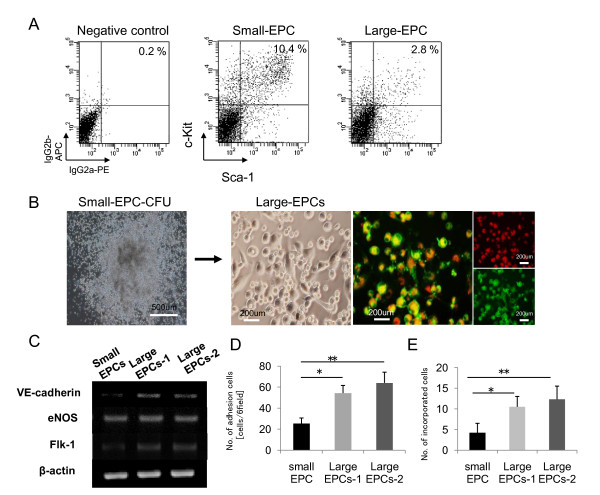
**Importance of small-EPCs as large-EPC-CFU-sprouting cells**. **(a) **Flow cytometry analysis of small-EPCs or large-EPCs after culturing for 10 days from freshly isolated bone marrow c-Kit^+^/Sca-1^+ ^lineage-negative cells (BM-KSL). **(b) **Secondary culture assay of small-EPC-CFUs from bone marrow mononuclear cells (BM-MNCs). Representative micrographs of small-EPC-CFUs from BM-MNCs before reseeding are shown on the left, and representative light and fluorescent micrographs of small-EPCs cultured secondarily in methylcellulose-containing medium are shown on the right. Secondary cultured cells were identified as double-positive cells due to 1,1'-dioctadecyl-3,3,3',3-tetramethyl-indocarbocyanine perchlorate-labeled acetylated-low density lipoprotein (acLDL-DiI) uptake (red) and BS-1 lectin reactivity (green). Small-EPCs could differentiate into large-EPCs. **(c) **The expression of vascular endothelial (VE)-cadherin, Flk-1, and endothelial nitric oxide synthase (eNOS) was measured in small-EPCs, small-EPCs-derived large-EPCs (Large EPCs-1), and large-EPCs (Large EPCs-2) by reverse transcription-polymerase chain reaction analysis. **(d) **Adhesion assay of small-EPCs, Large EPCs-1, and Large EPCs-2. *P < 0.05, **P < 0.01 versus small-EPCs. **(e) **Quantification of the number of cells incorporated into tubes in small-EPCs, Large EPCs-1, and Large EPCs-2. *P < 0.05, **P < 0.01 versus small-EPCs.

To determine whether small-EPCs are real immature cells, we performed FACS analysis on EPC-CFU-derived cells, which developed from fresh isolated BM-KSL (c-Kit^+^/Sca-1^+^/LNneg, purity of greater than 99.5%) cells. As shown in Figure 3a, we observed the higher population of KSL cells in small-EPCs, providing us a clue that small-EPCs contained actual progenitors. Therefore, to check whether small-EPCs can differentiate into large-EPCs, isolated small-EPCs were reseeded in methylcellulose-containing medium. PB-MNC-, BM-MNC-, or BM-KSL cell-derived-small-EPCs could differentiate into spindle-shaped cells, large-EPCs and could represent positivity of acLDL uptake and BS-1 lectin binding (Figure 3b, data not shown). To characterize small-EPCs-derived large-EPCs, we examined the gene expression of VE-cadherin, Flk-1, and eNOS; adhesion capacity; and incorporation potential of small-EPC-derived large-EPCs (large EPCs-1) compared with small-EPCs and large-EPCs (large EPCs-2). Gene expression profiles by RT-PCR revealed that large EPCs-1 strongly expressed VE-cadherin and Flk-1 compared with small-EPCs (Figure 3c). In the adhesion assay, the numbers of adherent small-EPCs, large EPCs-1, and large EPCs-2 were 23.2 ± 5.1, 52 ± 5.3, and 61.5 ± 8.3 per field, respectively (Figure 3d). In the tubular formation assay, more large EPCs-1 were incorporated into tubes compared with small-EPCs (Figure 3e). These results revealed that the large-EPCs derived from small-EPCs showed a higher potential of VE-cadherin expression, adhesion, and tube formation compared with those of small-EPCs, suggesting that small-EPCs might be more immature EPCs and be early EPCs, which could differentiate into large-EPCs.

### Kinetics of endothelial progenitor cell-colony-forming units in response to ischemia

**Figure 4:**
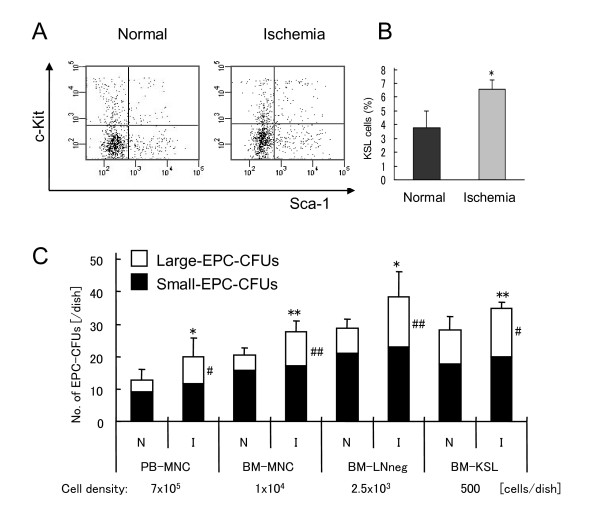
**Development of two types of endothelial progenitor cell colony-forming units (EPC-CFUs) in response to ischemia**. **(a) **Fluorescence-activated cell sorting (FACS) profiles of lineage-negative bone marrow cells (BM-LNneg). FACS analysis of BM-LNneg was performed by using rat IgG antibodies against mouse c-Kit and Sca-1. **(b) **The percentage of c-Kit^+^/Sca-1^+ ^lineage negative (KSL) population of BM-LNneg. Hindlimb ischemia induced KSL population in bone marrow. *P < 0.05 versus normal mice. **(c) **The frequencies of large-EPC-CFUs (white columns) and small-EPC-CFUs (black columns) from peripheral blood mononuclear cells (PB-MNCs), bone marrow mononuclear cells (BM-MNCs), BM-LNneg, and BM-KSL in normal mice (N) and hindlimb ischemic mice (I). Hindlimb ischemia increased the number of large-EPC-CFUs and total EPC-CFUs differentiated from PB-MNCs and bone marrow. *P < 0.05, **P < 0.01 versus total EPC-CFUs from normal mice. ^#^*P *< 0.05, ^##^*P *< 0.01 versus large-EPC-CFUs from normal mice. Hindlimb ischemia induced the differentiation of PB-MNCs and bone marrow.

EPCs play a critical role in restoration of ischemic diseases. To explore the effects of hindlimb ischemia on differentiation of BM into EPC-CFUs, we examined PB-MNCs and BM of hindlimb ischemic mice in EPC-CFA. This experiment could enable us to elucidate the roles of each EPC-CFU *in vivo*. First, hindlimb perfusion was evaluated by serial LDPI studies at day 5 after surgery. The ratio of blood flow between the ischemic and the normal limb was 0.19 ± 0.16, which was a significant difference compared with 0.98 ± 0.21 in the normal mice (data not shown). To explore the *in vivo *change in BM, we estimated the percentage of KSL population in BM by FACS analysis. The percentage of BM-LNneg did not change, but that of the KSL population in BM-LNneg was 6.6% ± 2.0% in ischemic mice, which was significantly increased compared with the normal mice: 3.8% ± 1.2% (Figure 4a, b). These data demonstrated that BM-KSL cells, which produced more EPC-CFUs, were induced by hindlimb ischemia. To check the differentiation capacities of EPCs from PB-MNCs and various fractions of BM-MNCs, the frequencies of EPC-CFUs from each population were counted. In all populations, the frequencies of large-EPC-CFUs and the ratios of large-EPC-CFUs were significantly increased in hindlimb ischemic mice (Figure 4c). These results indicated that hindlimb ischemia induced the differentiation of PB-MNCs and various populations of BM, implying that large-EPC-CFUs might play an important role in the restoration of ischemic diseases.

### Contribution of large-endothelial progenitor cells or small-endothelial progenitor cells to postnatal/adult neovascularization

**Figure 5:**
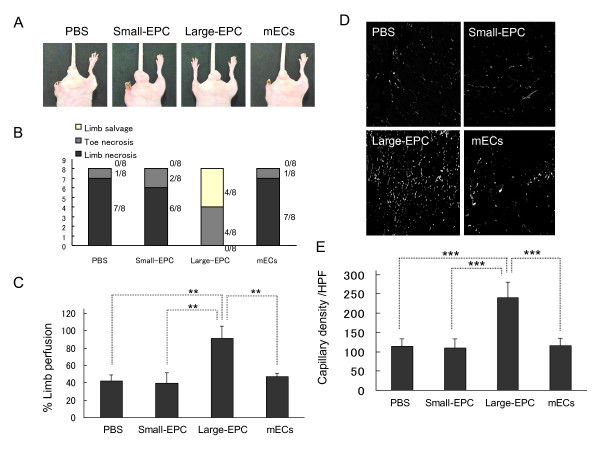
**Effect of two types of endothelial progenitor cell colony-forming units (EPC-CFUs) on neovascularization**. **(a) **Macroscopical observation of ischemic hindlimb 28 days after transplantation of large-EPCs and small-EPCs, which are derived from bone marrow c-Kit^+^/Sca-1^+ ^lineage-negative (BM-KSL) cells, and murine endothelial cells (mECs) into hindlimb ischemia. **(b) **The ratios of the above outcomes in each group. **(c) **Laser Doppler perfusion imaging showing reduction of blood flow at day 28 after surgery. Quantification data of blood flow as the ratio of perfusion in ischemic hindlimb to that in normal hindlimb are shown. In hindlimb ischemic mice, blood flow of hindlimb kept to be low in small-EPCs transplanted and other control groups at day 28 after surgery. Transplantation of large-EPCs recovered the limb perfusion in hindlimb ischemic mice. **P < 0.01 versus mice transplanted with large-EPCs (*n *= 8). **(d) **Representative images of iso-lectin B4-positive tissue 28 days after transplantation of large-EPCs, small-EPCs, and mECs into hindlimb ischemia. **(e) **The statistical data of (a). Transplantation of large-EPC-CFUs into hindlimb ischemia model enhanced neovascularization. ***P < 0.001 versus mice transplanted with large-EPCs (*n *= 8). HPF, high-powered field; PBS, phosphate-buffered saline.

To determine the functional importance of *in vivo *EPC status in a pathological situation, we transplanted large-EPCs or small-EPCs and murine ECs as controls into hindlimb ischemia models. As shown in Figure 5a, b, we observed limb salvage in large-EPC transplantation groups, although small-EPC, EC, or PBS transplantation groups did not operate as useful limb therapy cells. These macroscopical observations were further supported by monitoring of real blood flow by using a laser Doppler perfusion imaging system because the recovery of limb perfusion was significantly improved in large-EPCs transplantation groups only (Figure 5c) compared with those of small-EPC, EC, or PBS transplantation groups. Moreover, immunohistochemical analysis clearly showed that capillary density in large-EPC transplantation groups was markedly increased (Figure 5d, e), suggesting that large-EPC-CFUs are more functional EPC status for vascular regeneration *in vivo*.

## Discussion

EPCs can be classified into various differentiation levels in both circulating EPCs and tissue EPCs [16]. Here, we first defined *in vivo *EPC status by establishing the novel murine EPC-CFA, in which the colony-forming potential of EPCs at different differentiation levels can be assessed. We demonstrated, for the first time, that hindlimb ischemia induced onsets of large-EPCs, which might be the accelerated differential status of EPCs. The observation was further supported by an *in vivo *experiment in which transplantation of more mature large-EPCs into a hindlimb ischemia model enhanced neovascularization, implying the contribution of large-EPC-CFUs in a pathogenic situation as 'cells ready to operate'.

**Figure 6:**
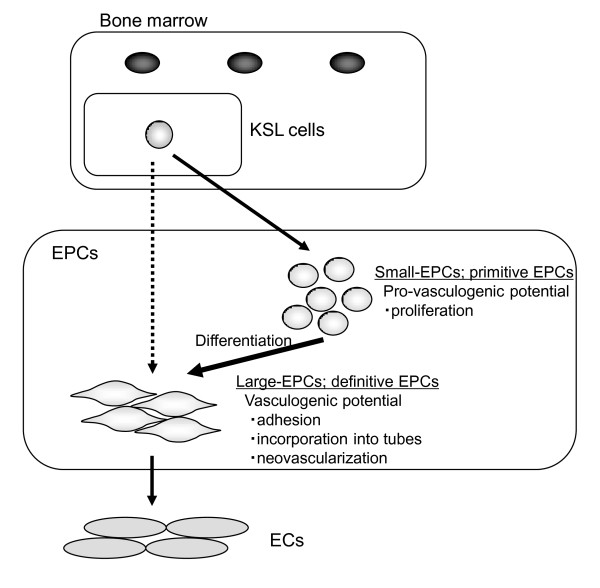
**Schematic model of endothelial progenitor cell (EPC) development**. In an endothelial progenitor cell colony-forming assay (EPC-CFA), three different stages of EPC development were classified: (1) stem cell stage as EPC-sprouting cells, (2) early stage of EPCs as large-EPC-CFU-sprouting cells, which contained mostly immature small cells, and (3) late stage of EPCs as functional EPCs, which contained mostly big spindle cells. EPC-CFU, endothelial progenitor cell colony-forming unit. EC, endothelial cell; KSL, c-Kit^+^/Sca-1^+ ^lineage-negative.

Previously, Hur and colleagues [20] reported that they found two types of EPCs - early EPCs and late EPCs - from a source of adult PB-MNCs; attached cells that appeared after 3 to 5 days of culture were defined as early EPCs, and cells that appeared in 2 to 4 weeks after plating were defined as late EPCs [20]. However, these classifications gave us some limitation for a full understanding of the EPC status. First, as these two types of EPCs were defined by different assays, two types of EPCs could not be assayed synchronously. Second, these assays failed to provide enough information about the differential cascade from immature stem cells, such as BM-KSL, into real EPC status. In our study, we redefined EPC status in response to a pathogenic situation. Small-EPC-CFUs had greater proliferative activity, suggesting that small-EPC-CFUs contained more immature clonogenic cells (KSL cells) derived from hematopoietic stem cells which preserve hemagioblastic potentials. Large-EPC-CFUs are sequentially differentiated from small-EPC-CFUs in response to ischemic signals (Figure 6). That is, small-EPC-CFUs are 'primitive EPCs' and large-EPC-CFUs are 'definitive EPCs'. Importantly, in regard to the vasculogenic potential *in vivo*, our study clearly demonstrated that transplantation of definitive EPCs (large-EPCs), not primitive EPCs (small-EPCs), markedly increased limb perfusion and capillary density and that small-EPC-CFUs have pro-vasculogenic potential and large-EPC-CFUs have vasculogenic potential, although early and late EPCs were reported to contribute equally to neovasculogenesis in a previous study [20]. Regarding the fact that small-EPCs did not show any therapeutic effect in Figure 5, we speculated three possibilities due to the low adhesion and incorporation potentials of small-EPCs: (a) transplanted small-EPCs could not survive in a hypoxic tissue environment, (b) transplanted small-EPCs could not differentiate into large-EPCs in a hypoxic tissue environment, and (c) transplanted small-EPCs could not show their function as secretion of growth factors in a hypoxic tissue environment.

Two types of EPC-CFUs represented distinct functional differences in both *in vitro *EPC colony study and *in vivo *EPC transplantation study. The adhesive potential and the incorporation into tubes formed by EC-like cells of large-EPCs were superior to those of small-EPCs, and small-EPCs had higher proliferation capacity than large-EPCs, which was consistent with the data on EPC-CFUs from BM-LNneg and BM-KSL (data not shown). In these points, definitive large-EPCs had similar functions to ECs compared with primitive small-EPCs. Besides, the secondary culture revealed that small-EPCs could differentiate into adherent cell, large-EPCs; in contrast, large-EPCs could not differentiate into round cell, small-EPCs (data not shown). These data showed that definitive large-EPCs are well-differentiated EPCs compared with primitive small-EPCs (Figure 1e). VE-cadherin is specifically expressed in adherent junctions of ECs and exerts important functions in cell-cell adhesion [28]. The different expression level of VE-cadherin between large-EPCs and small-EPCs might explain the better potential of adhesion, incorporation into tubes, and migration (data not shown) of definitive large-EPCs than those of primitive small-EPCs, which were consistent with our recent findings using human cord blood AC133^+ ^cells [29]. Gene expression profiles revealed that both EPC-CFUs were committed to endothelial lineage because both definitive large-EPCs and primitive small-EPCs expressed eNOS, Flk-1, and VE-cadherin, which are EC-specific markers [5]. However, both EPC-CFUs would be different from mature ECs in terms of colony formation capacity, tubular formation ability, and contribution of *in vivo *neovascularization, demonstrated by ischemia model, because ECs could not form colonies and did not have an effect on the restoration of blood vessels, and EPC-CFU-derived cells could not form tubes on Matrigel in a culture without ECs.

In this EPC-CFA, to compare the potentials to produce EPC-CFUs of three populations in BM (BM-MNCs, BM-LNneg, and BM-KSL), we calculated the numbers of cells producing one EPC-CFU in BM-MNC, BM-LNneg, and BM-KSL populations. It was revealed that one large-EPC-CFU was derived from 1.1 × 10^3 ^± 0.2 × 10^3 ^BM-MNCs or 3.6 × 10^2 ^± 1.1 × 10^2 ^BM-LNneg or 57 ± 34 BM-KSL (Figure 1b). One small-EPC-CFU was derived from 5.5 × 10^2 ^± 0.7 × 10^2 ^BM-MNCs or 1.2 × 10^2 ^± 0.2 × 10^2 ^BM-LNneg or 28 ± 3 BM-KSL (Figure 1b). These data demonstrated that BM-LNneg had 3- or 4.6-fold higher potential to produce large- or small-EPC-CFUs than BM-MNCs, respectively, and this suggested that more immature EPCs were contained mainly in the BM-LNneg population. In addition, it was demonstrated that BM-KSL had the highest potential to produce EPC-CFUs in any other populations in BM, and those potentials to produce large- or small-EPC-CFUs were 6.3- or 4.3-fold higher than BM-LNneg, respectively, and this suggested that immature EPCs were highly enriched in the BM-KSL population. In this study, using EPC-CFA, we determined that BM-KSL was the major population which highly enriched immature EPCs. We concluded, in this paper, that small-EPCs differentiated into large-EPCs because BM-KSL grew into small-EPCs about 5 days after plating and then those small-EPCs derived from BM-KSL could differentiate into large-EPCs in further culture. In our study, it remained unclear which niche component does small- or large-EPC differentiate from'. This should be definitely addressed in further issues.

In a clinical setting, the frequency of circulating EPCs serves as a biomarker for vascular function, and the number of circulating EPCs has been reported to be reduced in patients with diabetes mellitus or risk factors for coronary artery disease and to negatively correlate with the Framingham cardiovascular risk score [12-15]. Transplantation of EPCs into ischemic hindlimb or myocardial tissue improves organ function following new vessel growth [6-10]. Thus, EPCs play an important role in the restoration of ischemic vascular diseases. But essential molecular events that control the differentiation to EPCs and changes in EPCs in response to ischemia had not been clarified yet. Then we investigated the changes of EPCs in response to hindlimb ischemia in EPC-CFA and revealed that the population of KSL, which enriched immature EPC populations in BM, increased by ischemia. In previous studies, it was demonstrated that BM-derived EPCs were mobilized in response to tissue ischemia [26]. In this study, we showed, for the first time, that the ischemic signals could promote the differentiation of PB-MNCs, BM-MNCs, or BM-KSL cells into mature EPC-CFUs. Ischemia-induced differentiation into large-EPC-CFUs suggested that definitive large-EPC-CFUs as more mature EPCs might play an important role in the restoration of ischemic tissue, and this possibility was supported by the recovery of limb perfusion by transplantation of BM-KSL-derived large-EPCs into a hindlimb ischemia model compared with small-EPCs. In ischemic tissue, the expression of stromal cell-derived factor-1 (SDF-1) was induced by transcription factor hypoxia-inducible factor-1 (HIF-1) according to hypoxic gradients [27,28]. SDF-1 enhances differentiation of BM-derived c-Kit^+ ^stem cells into EPCs [29]. Thus, the EPC differentiation presented above might be promoted by SDF-1, which is induced by HIF-1 after ischemia.

## Conclusions

Our novel findings highlighted the actual status of EPCs via a redefinition of the differential stages of EPCs through BM-derived stem cells using our established murine EPC-CFA. The understanding of molecular cascades of EPC development from primitive small-EPC-CFUs to definitive large-EPC-CFUs will provide us some useful therapeutic advantages to solve the quantitative or qualitative problems for EPCs therapy.

## Abbreviations

acLDL: acetylated-low density lipoprotein; acLDL-DiI: 1,1'-dioctadecyl-3,3,3',3-tetramethyl-indocarbocyanine perchlorate-labeled acetylated-low density lipoprotein; BM: bone marrow; BM-KSL: bone marrow c-Kit^+^/Sca-1^+ ^lineage-negative; BM-LNneg: lineage-negative bone marrow cell; BM-MNC: bone marrow mononuclear cell; bp: base pairs; BrdU: bromodeoxyuridine; CFU-EC: colony-forming unit-endothelial cell; EC: endothelial cell; ECFC: endothelial colony-forming cell; EDTA: ethylenediaminetetraacetic acid; eNOS: endothelial nitric oxide synthase; EPC: endothelial progenitor cell; EPC-CFA: endothelial progenitor cell colony-forming assay; EPC-CFU: endothelial progenitor cell colony-forming unit; FBS: fetal bovine serum; FITC: fluorescein isothiocyanate; IMDM: Iscove's modified Dulbecco's medium; large EPC-1: large endothelial progenitor cell derived from small endothelial progenitor cell; PB-MNC: peripheral blood mononuclear cell; PBS: phosphate-buffered saline; PCR: polymerase chain reaction; PFA: paraformaldehyde; RT-PCR: reverse transcription-polymerase chain reaction; VE: vascular endothelial.

## Competing interests

The authors declare that they have no competing interests.

## Authors' contributions

ST and S-MK participated in study conception and design, collection or assembly of data (or both), data analysis and interpretation, and manuscript writing. TM, S-YJ, S-HL J-HL, and HM participated in collection, assembly, analysis, and interpretation of data. TA drafted and revised the manuscript. All authors read and approved the final manuscript.
